# Analysis of Graphic Perception Education for Young Children Based on Fuzzy Clustering Analysis

**DOI:** 10.1155/2022/8046713

**Published:** 2022-06-21

**Authors:** Jie Gui, Joohyun Suh

**Affiliations:** ^1^Jiangsu Vocational College of Electronics and Information, Huaian, Jiangsu 223003, China; ^2^Department of Family Welfare, Sangmyung University, Jongno-gu, Seoul 03016, Republic of Korea

## Abstract

Geometric ability includes elements of identification, conceptualization, combination, drawing, and reasoning, and graphic perception is an important part of it. Kindergarten science education includes geometry instruction. Children are guided to perceive the relationship between shapes and figures through direct perception, first-hand experience, and practical operation through concentrated educational activities, and form image-concrete thinking over time, enhancing their perception and experience of the relationship between shapes in the objective world, and accumulating certain mathematical perceptual experience. Clustering is a branch of unsupervised pattern recognition that is very useful. Fuzzy clustering, which establishes the uncertainty description of samples to categories and can objectively reflect the real world, has become the mainstream of cluster analysis research. The graphics perception education of children is investigated using fuzzy clustering analysis. The main topic of this paper is how to apply children's graphics to the design of children's educational institutions and open up new creative perspectives for the design of children's educational institutions. The method of graphic perception education: perception education for preschool children is proposed based on the multichannel characteristics of preschool children's aesthetic perception and with reference to the theory of perception. The experimental results show that the improved algorithm reduces segmentation time by 171.48 s when compared to the traditional FCM algorithm for both noisy and high-quality images and that it is significantly faster than the FCM algorithm in terms of segmentation speed. As a result, the model construction of a set of children's graphic perception education for the cognitive characteristics of the age group children can provide corresponding references and references for related topic research.

## 1. Introduction

The abstraction and generalization of shape are known as geometry. In the process of human understanding of nature, it appeared as a symbolic system before language and words [1]. Children's geometry education is an important part of their mathematics education, and the living of geometry education encourages children's perception and experience of graphical relationships in the objective world, allowing them to accumulate perceptual experience in mathematics [2]. The ability to recognize and combine plane figures is reflected in two main areas in early childhood [3]. Learning graphics properly aids children in developing a correct understanding of life and the world around them, as well as their curiosity, curiosity, and interest in geometry and mathematics [4]. It also aids in the development of children's thinking abilities and qualities, which will aid in their future mathematics learning in elementary school [5]. Living conditions are improving, and parents are becoming more aware of the importance of having their children educated in graphics, indicating that children's art education is entering a new era [6].

People communicate with the outside world through their senses. Children, in comparison to adults, rely on their senses to gather information about the world around them [7]. People always try to find features that can describe a new object or phenomenon in order to compare it to known objects or phenomena to see if they are similar according to some fixed criteria and rules in order to recognize it or understand it [8]. Only certain parts or regions of an image are of interest in the study and application of images, and these parts are referred to as the target or foreground, whereas other parts are referred to as the background and generally correspond to specific and unique regions of the image [9, 10]. Perceptual design and graphic design are being studied together in order to integrate perceptual design and children's art educational spaces. The main goal is to bring children closer to the art education space, reflect the educational function of the environment, mobilize children's perceptions through their visual, tactile, olfactory, and auditory senses, and promote children's enthusiasm for knowledge exploration [11].

Geometric thinking skills, which include elements of identification, conceptualization, combination, drawing, judgment, reasoning, and presentation, are an important part of geometric abilities [12]. The targets in an image must be separated and extracted before they can be identified and analyzed. It is possible to further measure the targets and use the images on this basis. Cluster analysis [13] in kindergartens for geometric educational activities helps children perceive geometric features through seeing, touching, thinking, guessing, and trying and deepens and consolidates their knowledge of geometric figures [14]. Color images, on the other hand, have unquestionably assumed a dominant position in the new century, thanks to technological advancements and lower hardware costs. Color images play an important role in people's daily lives and technological applications because of their visual properties and rich color information. Young children learn inexhaustibly and can achieve great success in their future learning if they are equipped with the ability to transfer and construct knowledge. The following are the paper's unique features.

1. Applying perceptual design to graphics, children's educational space embodies space can promote children's art education through children's sense of hearing, touch, smell, and vision to promote children's learning process.
2. To find the inner connection between fuzzy cluster analysis and children's geometric graphic education activities, and to dig, filter, and refine the mathematical elements in the resources that are suitable for children's geometric graphic education activities.
3. To address the shortcomings that the fuzzy C-mean clustering image segmentation [15] algorithm does not consider the different degrees of the contribution of each dimensional feature to the clustering and easily falls into local optimum, an improved fuzzy clustering algorithm based on kernel function is proposed.

This paper's research framework is divided into five major sections, which are listed below. The first section of this paper introduces the research background and significance before moving on to the paper's main work. The second section covers graphic perception training for young children as well as work on fuzzy clustering analysis. The third section of the paper introduces the conceptual direction of graphic perception education as well as the steps for implementing perceptual strategies in graphic education so that readers can gain a better understanding of the concepts of graphic perception education for young children using fuzzy cluster analysis. The thesis's fourth section concludes the description of fuzzy clustering analysis' application in early childhood graphic perception education by looking at two aspects: the analysis of fuzzy C-mean clustering image segmentation algorithm and the analysis of improved kernel function-based fuzzy clustering algorithm. The final section of the paper is a summary of the entire project.

## 2. Related Work

### 2.1. Children's Graphic Perception Education

Geometry is an important part of a child's development and is one of the fundamental components of their mathematics education. Geometry education activities in kindergarten refer to the geometry education activities that teachers plan and implement purposefully, planned, organized, and systematically within the time constraints of normal kindergarten teaching activities, in accordance with the rules of children's physical and mental development, as well as the characteristics of mathematical cognitive development. Through the transformation and application of geometric figures, as well as the use of symmetrical features to analyze mathematical situations, children can analyze the characteristics and properties of two- and three-dimensional geometric figures ad use visual and spatial reasoning, as well as geometric models, to solve problems.

The applications proposed by Tai and Ledai require teachers to arrange and organize the content in a way that integrates with other artistic activities such as music, dance, and literature, as well as with children's life experiences, in a way that helps children to generate “common experiences” and “complementary experiences” [16]. Li et al. proposed the five developmental levels for children, which became the most influential theory for the geometry curriculum in the United States and has been refined in subsequent developmental studies [17]. Feng and Chen argued that art appreciation works should be appreciated in a sequence from abstract to concrete and then from abstract to concrete. Works with strong emotional expression are the primary consideration in selecting works [18]. Rajkumar et al.'s developmental levels of graphic perception education describe the process of children's geometric thinking and the characteristics of children's geometric thinking at each stage, not just how much geometric knowledge children have mastered [19]. Shakeri et al. argue that preschool children's preferences for color and expressive style of artworks reflect their preference for aesthetic features [20].

Exploring the characteristics and patterns of children's graphic perception not only helps teachers to better understand the importance of teaching children's geometry but also helps them to grasp the characteristics and patterns of children's graphic perception and to gain a concrete and in-depth understanding of the developmental process of children's geometric thinking skills.

### 2.2. Fuzzy Cluster Analysis

In recent years, domestic research on children's graphic perception abilities has begun to emerge, primarily from the perspective of child psychology. Further research in the areas of fuzzy cluster analysis and multicultural perspectives is required. Children learning mathematical knowledge is only the surface manifestation of development in mathematics education; the key is to encourage the development of children's thinking structures through the learning process. The basis of target representation is image segmentation based on fuzzy cluster analysis, which has a significant impact on feature metrics. Image segmentation and goal representation, segmentation-based feature extraction, and parameter measurement, on the other hand, reduce the original image to a more compact form, allowing for higher-level image analysis and comprehension.

Goyal et al. proposed clustering algorithms for image segmentation because the subjective nature of human vision makes images more suitable for fuzzy processing [21]. Pham et al. used fuzzy entropy to analyze the homogeneity of images to obtain initial major homogeneous regions and then grouped pixels with similar colors in these regions to prevent oversegmentation [22]. Berikov proposed that fuzzy should be used in image segmentation processing. Also, the absence of patches in training sample images requires unsupervised analysis, and fuzzy clustering satisfies both requirements, thus becoming a powerful research and analysis tool in image processing [23]. Mlr et al. proposed a combination of the histogram and fuzzy C-mean clustering. This method obtains all possible uniform regions in a color image by histogram thresholding and then improves the tightness of the regions by fuzzy C-mean clustering [24]. Jia and Zhang used an automatic histogram analysis method to obtain the peaks and troughs of the histogram by automatic histogram analysis, then performed fuzzy clustering according to the determined number of clusters, and then used the distance between adjacent troughs in the histogram The mean value between adjacent troughs in the histogram is used as the initial clustering center.

Unsupervised classification, also known as cluster analysis, is the process of distinguishing and classifying things according to certain requirements and rules. In this process, there is no a priori knowledge about the classification [25], only the similarity between things as a criterion for class classification. Through education in recognizing geometric figures, combining and dividing geometric figures, and drawing geometric figures, children can get an initial understanding of the characteristics of shapes, learn to spell and draw them creatively, stimulate their interest in learning mathematics, and develop their ability to think about concrete images.

## 3. Thoughts on Education of Children's Graphic Perception Based on Fuzzy Cluster Analysis

### 3.1. Conception Direction of Graphic Education

Perception is an active process based on the relationship between a person and the outside world. From the day of birth, a person can spontaneously acquire knowledge about the inner self and the outer environment through the different senses of the body [26]. The education of graphic perception needs to be diffused and recorded, taking the text closest to the design focus to further deepen it. By splitting and reorganizing the various visual elements, the information and ideas that are wanted to be conveyed are translated into concrete graphics and then clearly communicated to the viewer [27]. For the given number of categories *c*, fuzzy weighted division matrix *U*, and weighted coefficient matrix *W*, the fuzzy division entropy of the sample set *X* is defined as

(1) $$\begin{array}{c} H\left(U;c\right)=-\frac{1}{N}\sum_{k=1}^{n}\sum_{i=1}^{c}w_{k}u_{ik}\ln\left(u_{ik}\right). \end{array}$$

**Figure 1:**
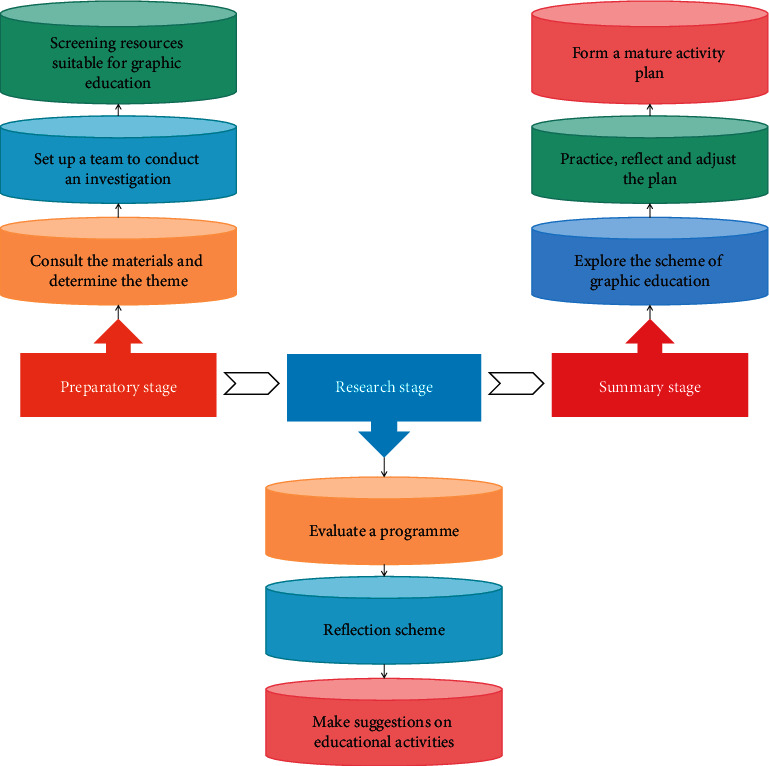
Research flow chart of kindergarten profit graph education activities.

The activities were adjusted according to the specific practice, and educational activities were suggested to promote the cognitive development of children in mathematics. The specific flow of the study is shown in Figure 1.

First, depicting reality is a depiction and replication of what exists in reality, a reproduction of the real thing. Also, parents say that the teaching environment is most likely to make a first impression. Teachers select appropriate domestic and international paintings based on the age, preferences, and appreciation of children and the “principle of difference” in their aesthetic psychology. Therefore, the composite index value is not equal to the simple sum of the indicators but a weighted summation relationship, namely,

(2) $$\begin{array}{c} S=\sum_{i=1}^{n}w_{i}f_{i}\left(I_{i}\right),\;i=1,2,\ldots ,n, \end{array}$$

where *f*_*i*_(*I*_*i*_) is some measure of *I*_*i*_, and *w*_*i*_ is the weight value of each index.

The integration of cluster analysis into geometry educational activities can make geometry vivid and visual, stimulate children's interest, bring them sensory stimulation from many aspects, and mobilize children's multiple senses to participate in activities [28]. For a given number of categories *c*, fuzzy weighted division matrix *U*, and weighted coefficient matrix *W*, the modified division fuzziness of the sample set *X* is defined as

(3) $$\begin{array}{c} \text{MPF}\left(U;c\right)=\frac{PF\left(U;c\right)}{H\left(U;c\right)}. \end{array}$$

**Figure 2:**
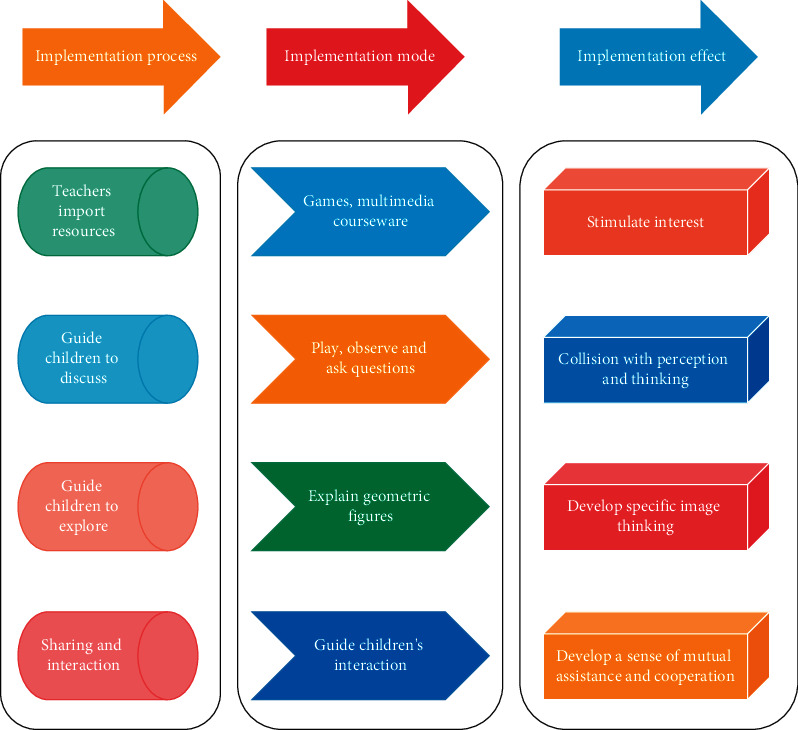
Flow chart of geometric figure teaching activities in kindergarten by using perceptual education.

Feature extraction [29] is the generation of useful new features from the original features through some transformations, whereas feature selection is the selection of features that help to distinguish different objects from a set of candidate features. Perception is the knowledge of various other elements of things and a complete, three-dimensional, and multidimensional overall grasp of the shape, color, space, light, tension, and texture of things [30]. Children's senses are generated through their senses as a process of representation of different stimuli of the external environment and a reflection of the individual characteristics of things. The designed activity program places children at the center of the activity, incorporates perceptual education into the teaching process, and encourages children to participate in teaching activities in a fun way. The flowchart of teaching activities of geometric figures using kindergarten perceptual education is shown in Figure 2.

Secondly, artistic abstraction refers to the abstract expression of things; it is a kind of art form which is detached from the natural appearance. Generally speaking, artistic abstraction expresses subjective attitude through color and shape. The teacher needs to analyze the formal elements, spatial characteristics, and colors of the painting in a comprehensive, in-depth, and detailed manner in order to identify the forms that fit the definition of the “perception” strategy. This step is usually combined with the selection of the appropriate similarity measure and the establishment of a criterion function, followed by the grouping of samples according to whether they are similar to each other or not. For any sequence that iterates and eventually converges, the speed of convergence increases significantly and the number of iterations decreases significantly when the initial value of the iteration is close to the final convergence result. Children can understand the characteristics of graphs through experience, which is a favorable condition for teaching graphs. Also, this feature can explain the types of errors children make: similar graphs, similar objects, and confusing names. A higher affiliation is surrounded by a lower affiliation of the same category. Based on this understanding, a new objective function can be introduced:

(4) $$\begin{array}{c} J_{m}\left(U,V\right)=\sum_{k=1}^{n}\sum_{i=1}^{C}{\left(u_{ik}\right)}^{m}{\left(d_{ik}\right)}^{2}+\frac{\beta }{N_{R}}\sum_{k=1}^{n}\sum_{i=1}^{C}{\left(u_{ik}\right)}^{m}\sum_{j\in Nk}{\left(1-u_{ij}\right)}^{m}. \end{array}$$

Finally, spatial constructions were mostly made by stacking geometric shapes, and spatial constructions were made by matching the shapes and colors of geometric shapes. Compared to circles, squares, and triangles, circles accounted for the majority of children's scribbles, with a few concentrated in squares or triangles. Given a dataset, each clustering algorithm can generate a division regardless of the presence or absence of a clustering structure. Moreover, different methods usually produce different clustering results. Even for the same clustering algorithm, differences in parameters or the order of input samples may affect the final result. The urban kindergarten teacher did not teach the children geometry during this time, so the difference caused by the time factor is negligible.

### 3.2. Steps of Applying Perception Strategies in Graphic Education

**Figure 3:**
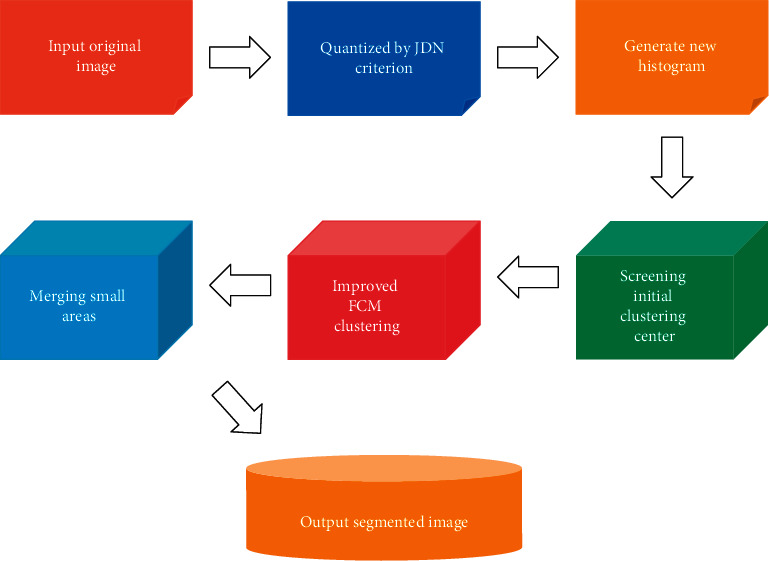
Schematic diagram of color image segmentation process.

When incorporating perceptual education in kindergarten geometry education activities, it is important to pay attention to the program design and the flow of activities according to the laws of kindergarten education and teaching activities and children's development. Children show disappointment in front of works with which they are very familiar, but they are indifferent when something completely unfamiliar is put in front of them. Only those things that are different from what they are familiar with, but can be seen to have some connection to them, are the ones that really appeal to them. The clustering method is to obtain homogeneous regions by manipulating the pixel features of images, while region growth is to divide regions by combining pixel features of the image domain and spatial information. The color image segmentation process in this paper is schematically shown in Figure 3.

First of all, choose paintings with strong musical expression. Geometry education activities applied to cluster analysis in kindergarten take the familiar living resources of young children as the carrier and the process of playing games as the main form of educational activities, so that children can increase their perception of geometric figures in the process of fun and education. Many masters of painting, both ancient and modern, have created numerous classic works, but not all of them are suitable for art appreciation activities using perceptual strategies. Because the unit used in each feature is incomparable, its size can represent the characteristics of the sample in that feature. Based on the principle of feature balance, that is, the contribution of each feature to the classification is essentially equally important, let the balance coefficient be *r*_*j*_, which is calculated as follows:

(5) $$\begin{array}{c} r_{j}=\frac{\max\left\{\sum_{i=1}^{c}p_{il},l=1,2,\ldots ,s\right\}}{\sum_{i=1}^{c}p_{il}},\;j=1,2,\ldots ,s. \end{array}$$

The whole technique optimizes the points that may be pixels in the whole edge of the image, taking into account the overall variation of the large area, so as to obtain the most suitable set of edge points. The image is segmented using the standard FCM algorithm to obtain the best division matrix *U* and the objective function value *J*_FCM_, and then the following function values corresponding to this division matrix *U* are calculated:

(6) $$\begin{array}{c} J_{\text{add}}=\frac{1}{N_{R}}\sum_{k=1}^{n}\sum_{i=1}^{c}u_{ik}^{m}\sum_{j\in N_{k}}{\left(1-u_{ij}\right)}^{m}. \end{array}$$

Then use the following equation to calculate the coefficient *β*:

(7) $$\begin{array}{c} \beta =\frac{J_{\text{FCM}}}{J_{\text{add}}}. \end{array}$$

Children can master eight shapes in terms of difficulty. From easy to difficult, the order is circle, square, triangle, rectangle, semicircle, trapezoid, rhombus, and parallelogram. In the splitting phase, the homogeneity criterion is used to divide image regions into many homogeneous regions, and in the merging phase, adjacent similar regions are merged into one region. The instability of children's pattern recognition and the inattention to children's exams can affect the development of children's pattern recognition ability. As we know from the role of standard deviation in mathematical statistics, standard deviation describes the degree of concentration and dispersion of data. So the degree of class separation can be measured by the standard deviation of the clustering prototype, which is calculated as follows:

(8) $$\begin{array}{c} d_{ij}=\sqrt{\sum_{i=1}^{c}{\left(p_{ij}-\bar{p_{j}}\right)}^{2}},\;j=1,2,\ldots ,s. \end{array}$$

Second, timely and appropriate display of paintings is the second step in implementing the perception strategy. Based on the laws of children's physical and mental development, children are provided with objects, pictures, videos, etc. It is easy to use the surrounding life resources for visual observation and direct manipulation in educational activities and can be explained concisely in a language that children can understand to promote their correct perception of geometric figures. However, there is a mixture of nonestablishment and establishment in children's doodle investigations on a piece of paper, so it cannot be concluded that they are completely ignorant, but only that the degree of understanding of the investigation varies at this moment. Fuzzy clustering segmentation generally fails to effectively use the information of the spatial relationship between image pixels, which easily leads to discontinuity in the segmented region. In another segmentation, the number of categories may be incorrect, and there is often the possibility of oversegmentation. Let *X*={*x*_1_, *x*_2_,…, *x*_*n*_} be a collection of sample data mapped from the input pattern space *R*^*s*^ to the feature space *R*^*p*^ by a nonlinear transformation *φ*(•). The objective function for partitioning the data set into classes is

(9) $$\begin{array}{c} J_{m}=\sum_{i=1}^{c}\sum_{j=1}^{n}u_{ij}^{m}{\left\|\varphi \left(x_{j}\right)-\varphi \left(v_{i}\right)\right\|}^{2}. \end{array}$$

Therefore, multiple clustering centers are selected as seed regions. Using these seeds as starting points, different strategies and methods are used to classify the neighboring pixels around them that satisfy their feature conditions. Generally, after the clustering is completed, the segmentation results need to be processed by some merging classes so that the final segmented regions are meaningful. Let *K* be the kernel function defined on the dataset, which is defined by an implicit mapping:

(10) $$\begin{array}{c} K\left(x,y\right)=\langle \varphi \left(x\right),\varphi \left(y\right)\rangle ,\varphi :X⟶F. \end{array}$$

Finally, mobilizing children's sensory systems such as hearing, smell, taste, and touch to improve the appreciation of drawing is the third step in implementing the perception strategy. Children are fully guided to develop a strong interest in participating in geometric educational activities by creating contexts, providing manipulative materials, and designing games, with a focus on stimulating their interest in observation, thinking, and manipulation, making the process of educational activities vivid and interesting, and enhancing their desire to explore geometric shapes. The original histogram is recalculated using the JND color model with the minimum visible difference in human vision, and the three-dimensional axes of the original image histogram are sampled without affecting human vision to obtain new histogram pixel values, whether children's planar geometric figure recognition and combination abilities are positively or negatively correlated. Regardless of which research is conducted, the findings should ultimately guide practice, and the findings should assist kindergarten teachers in better understanding the characteristics and trends of young children's graphic perception.

## 4. Application Analysis of Fuzzy Cluster Analysis in Children's Graphic Perception Education

### 4.1. Analysis of Image Segmentation Algorithm Based on Fuzzy C-Means Clustering

**Figure 4:**
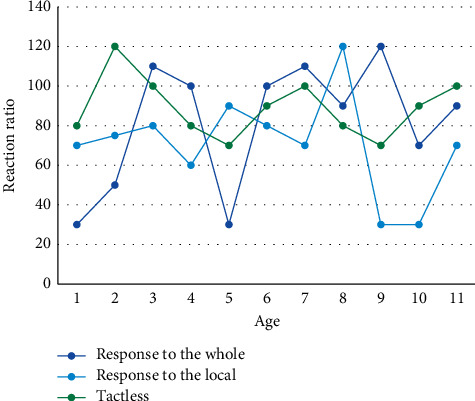
Ratio of responses of subjects of different age groups to global and local characteristics under the condition of free attention.

The FCM algorithm is used for image segmentation by fuzzy clustering pixels with consistent attributes in an image and then calibrating the pixels in each class. In most cases, the given dataset consists of several samples. Each sample is multidimensional, and each dimension corresponds to a feature or attribute value. No matter how the objective function is defined in the FCM algorithm, there is a corresponding clustering prototype corresponding to it, so the convergence speed and even the clustering effect necessarily depend on the initial division. Since child subjects cannot respond as quickly as adult instructions, the response rates of subjects of different age groups to global and local features in the free attention condition are shown in Figure 4.

**Figure 5:**
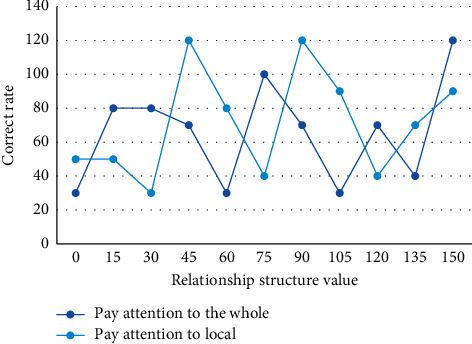
5-year-old subjects' attention to the whole is influenced by the structural relationship.

First, the pixels of the images are used as sample points of the data set, and the features of the pixels are the features of the grayscale images; i.e., the grayscale images are used as sample points. The test of children's planar geometry combination ability includes two parts. The characteristic of the combined audiovisual animation is that the animation content is a reproduction of the painting content, which gives life to the painting and makes the frozen moment flow. Since only the grayscale information of pixels is used in the grayscale threshold segmentation, the grayscale histogram can be used for clustering instead of the pixels of the image. However, because the statistical information of the original statistical histogram is scattered, the direction of the histogram curve may not be very clear and the peaks and valleys cannot be well selected for image segmentation. Therefore, the original histogram must be smoothed before it can be used in the subsequent segmentation center selection process. A further significance test was conducted to examine the difference between responses focusing on the whole and those focusing on the local. Setting up experiments in which 5- versus 10-year-old subjects were influenced by structural relations when paying attention to the whole, the experimental results are shown in Figure 5.

**Figure 6:**
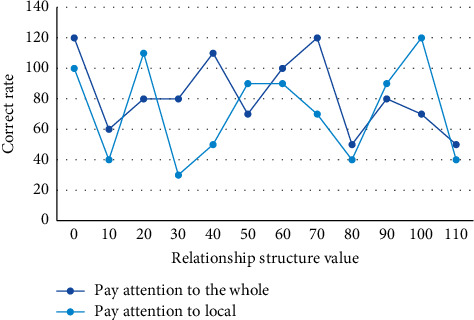
The 10-year-old group was influenced by the structural relationship when paying attention to the whole.

Second, the image is segmented using the difference between the background and the object as shown in Figure 6, taking a complete circle and dividing it into two semicircles, three obtuse sectors, and four right-angled sectors. To prepare for perceptual drawing, creatively recreate situations based on the teacher's picture composition using manipulable material materials, similar structural forms, and emotions. The number of samples in the grayscale domain is a fixed value independent of image size and equal to the number of grayscales in grayscale images once the grayscale of the image is known. If the image to be segmented has a clear difference between the background and the segmented object, i.e., if the histogram has a clear threshold that can segment the image into several separate parts, this method allows for quick and simple segmentation.

Finally, the pixel values of one or more image dimensions are counted to produce a histogram of pixel statistics for the image's dimensions. The histogram will show distinct peaks in homogeneous regions in an image with similar colors. The number of samples for clustering will be greatly reduced, the complexity of the computational matrix will be reduced, and the efficiency of the general-purpose algorithm for image segmentation will be increased if the pixels of an image are clustered with grayscale histograms instead. Geometry is vibrant, and so should the educational activities that accompany it. It is critical to fully explore children's interest in geometry and to allow them to enjoy geometry educational exploration activities through games. The juxtaposition of reality and imagination characterizes the combination of art appreciation and physical objects, with the same image highlighting the painting's unique color matching, form, and other artistic aesthetics. Set a color merging range after determining the central pixel values of several clusters. The color distance between all pixels in the image and these central pixels should then be calculated. The colors are merged if they are within the range; otherwise, they are identified as new cluster centers, and so on, until all pixels have been processed.

### 4.2. Analysis of Improved Fuzzy Clustering Algorithm Based on Kernel Function

Classical clustering algorithms directly use Euclidean distance as a similarity measure, and close samples in the sample space are clustered together. However, the commonly used edge operators are wide edges, so the obtained edges must be refined to ensure accurate color values are obtained. Therefore, the improved kernel function-based fuzzy clustering algorithm can map the samples in the input sample space to a high-dimensional feature space in which the nonlinear learning problem may become a linear learning problem. In this study, the mean scores and standard deviations of basic plane geometry figure recognition of 200 children in three age groups of elementary, middle, and large classes were statistically analyzed, as shown in Table 1.

**Figure 7:**
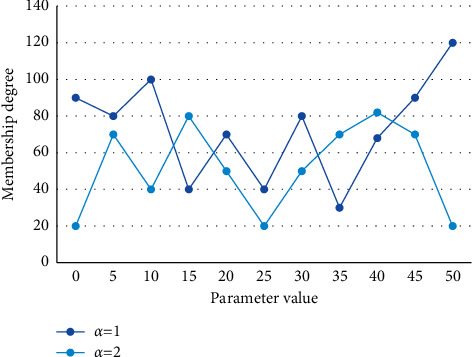
The membership histogram of each sample belonging to the first class when the *α* value takes some representative values.

The clustering process is guided by making the affiliation values of the labeled samples as close as possible to the labeled affiliation values, provided that the structure of the dataset is discovered in the unsupervised part of the previous section. The histogram of the affiliation of each sample belonging to class 1 when the *α* value is taken as some representative value is shown in Figure 7.

**Figure 8:**
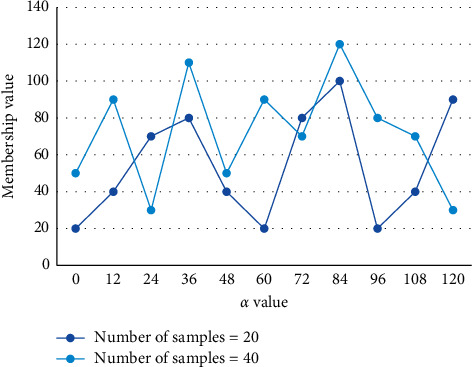
Changes in membership values of different supervised samples with the increase of *α* value.

First, an image is viewed as a continuum of closely connected, interrelated pixels. Its individual features are part of the global features, affecting the values and features of other pixels on the edges, not in isolation. The cluster center values and affiliation of each sample to each cluster center value are corrected through iterative operations of the fuzzy classification matrix and the cluster center matrix to finally find the categorical features contained in the sample data set, thus classifying each sample into the category with the greatest affiliation. Children's understanding of geometric figures is significantly higher than their ability to name them correctly, and children's representations of figures are influenced by verbal expressions, which reveal inconsistencies between perception and expression. Subjects should be more accurate when paying attention to the whole than to the part, and the response to the whole should not vary with structural relations, whereas the part's response to contradictory structural relations should be worse than unrelated structural relations or even worse than consistent structural relations, and the response rate to the whole should be higher during free attention. The objective function is divided into two parts, similar to the improved kernel function-based fuzzy clustering algorithm FCM algorithm, but the latter part only contains labeled samples. The variation of the affiliation values of different supervised samples with increasing values of *α* is plotted in Figure 8.

Second, the color difference of each region to be segmented is small, but the difference between regions is large. From the concept of classification, the distance between classes is the smallest and the distance between classes is the largest. There is a positive transfer between children's perception of plane geometry and their perception of geometry. So the whole computational process is a process of iteratively modifying the clustering centers and classification matrix, which can converge from any given initial point along an iterative subsequence to the local minima or saddle points of its objective function. The inclusion of childlike graphics in the design of children's educational institutions helps children to acquire cognition, which in turn helps them to better understand their external environment. This proves that children can notice the front-back position and shading relationships of objects through visual perception and also want to represent them through drawing. Therefore, the difference between the gray value of the gray image and the gray value of the seed point is used as a criterion, and the rule for the end of growth is that all pixels grow completely and the distance between classes is greater than a certain threshold. In the improved algorithm, if both 2-DHs are from the original image, their binary groups will be distributed on the diagonal of the plane, so the segmentation of the improved algorithm is consistent with that of the standard FCM algorithm. The performance comparison of the two segmentation algorithms is shown in Table 2.

In summary, the segmentation time of the improved algorithm is significantly reduced by 171.48 s compared to the traditional FCM algorithm, whether it is used to segment noisy images or images with better quality, and thus significantly outperforms the FCM algorithm in terms of segmentation speed.

Finally, *m* sample instances are randomly selected from the training set. The correlation between each feature of each sample and the class is obtained by the difference between the selected samples and the two nearest neighbors belonging to the same class and different classes, and then the average value is taken as the weight of each feature. Children's perception of plane geometry and geometry goes through a process of pairing–identifying–naming from perceiving the external features of geometry to being able to name it. In order to get the best clustering results, the Lagrange multiplier method is used to obtain the affiliation function and the clustering prototype update formula, so as to obtain the alternatively optimized image segmentation results. The image is segmented into disjoint blocks, and then, the features of the blocks are judged to be consistent, and if they are consistent, they are merged, otherwise, the inconsistent blocks are segmented into blocks as parent blocks and judged again, and the process is repeated until all the features within the blocks are consistent, as well as the features between the blocks are different.

## 5. Conclusions

Children's artworks reflect their perceptions of the outside world and express their inner feelings and thoughts. Art plays an essential role in preschool education as a fundamental subject. The ability of children to combine plane geometry is primarily at the fragmented combination and image stages, with some children at the precombination and shape combination stages. Preschoolers are in the early stages of self-awareness and understanding of the world, and their sense organs are rapidly developing. The development trend in children's thinking about the recognition of plane geometric figures is from visual to analytical, from focusing on the overall visual image to considering the figure's characteristics and concrete image thinking. Image segmentation is the problem of classifying the pixels of an image, and fuzzy theory has been found to have a good ability to describe image uncertainties. Graphics can help preschool children develop their visual system perceptual abilities by influencing their ability to perceive basic shapes, spatial relationships, colors, and visual memory. This research aims to improve children's perceptions of geometric figures, develop their unique figurative thinking, pique their interest in educational graphic perception activities, investigate the methods and paths of using fuzzy clustering analysis to develop educational geometric figure activities in kindergartens, and expand the ideas of developing garden-based curriculum resources in kindergarten science. As a result, the child-centered analysis of young children's graphic perception education proposed in this paper can combine the characteristics of preschool children's perceptual development and fuzzy cluster analysis to help graphic education implementers design a more popular curriculum for children and successfully carry out graphic perception teaching activities.

## Figures and Tables

**Table 1:** Average score and standard deviation of basic plane geometry recognition of children of all ages.

	Reception class	Middle class	Big class
Triangle average	3.24	5.98	10.54
Triangle standard deviation	0.45	1.65	2.77
Circular average	2.78	4.26	7.84
Circular standard deviation	0.27	1.27	1.85
Rectangle average	1.73	3.76	5.98
Rectangle standard deviation	0.17	0.94	1.26

**Table 2:** Comparison of segmentation results of Lena image polluted by the noise by two algorithms.

Algorithm	FCM	Improved algorithm
Split time (s)	189.26	17.78
Cluster center	24, 88, 137, 176	24, 90, 138, 177

## Data Availability

The data used to support the findings of this study are available from the corresponding author upon request.
